# Quadriceps or triceps surae proprioceptive neuromuscular facilitation stretching with post-stretching dynamic activities does not induce acute changes in running economy

**DOI:** 10.3389/fphys.2022.981108

**Published:** 2022-09-29

**Authors:** A. Konrad, M. Tilp, F. Stöcker, L. Mehmeti, N. Mahnič, W. Seiberl, D. G. Behm, F. K. Paternoster

**Affiliations:** ^1^ Institute of Human Movement Science, Sport and Health, Graz University, Graz, Austria; ^2^ Associate Professorship of Biomechanics in Sports, Technical University of Munich, Munich, Germany; ^3^ School of Human Kinetics and Recreation, Memorial University of Newfoundland, St. John’s, Newfoundland and Labrador, Canada; ^4^ Prevention Center, Department of Sport and Health Sciences, Technical University of Munich, Munich, Germany; ^5^ Department of Human Sciences, Institute of Sport Science, Universität der Bundeswehr München, Neubiberg, Germany

**Keywords:** stretching, running performance, running biomechanics, performance potentiation, plantar flexors

## Abstract

Previous studies reported that both a more compliant quadriceps tendon and a stiffer Achilles tendon are associated with better running economy. While tendon stiffness can be decreased by a single bout of proprioceptive neuromuscular facilitation (PNF), post-stretching dynamic activities (PSA) can counteract the potential stretch-induced force loss. Thus, the purpose of this study was to investigate if a single, moderate duration, (4 × 15 s), bout of PNF stretching of either the quadriceps or triceps surae muscles followed each by PSA, causes either an improvement or impairment in running economy. Eighteen trained male runners/triathletes visited the laboratory five times. The first two visits were to familiarize the participants and to test for maximal oxygen consumption (VO_2_max) respectively. The further three appointments were randomly assigned to either 1.) quadriceps PNF stretching + PSA or 2.) triceps surae PNF stretching + PSA or 3.) no stretching + PSA. Following the interventions, participants performed a 15-min run on the treadmill with a speed reflecting a velocity of 70% VO_2_max to assess oxygen consumption (i.e., running economy) and running biomechanics. Our results showed neither a difference in oxygen consumption (*p* = 0.15) nor a change in any variable of the running biomechanics (*p* > 0.33) during the steady-state (i.e., last 5 min) of the 15-min run. Athletes can perform moderate duration PNF stretching of the quadriceps or triceps surae + PSA prior to a running event, without affecting running economy. Future studies should emphasize long-term training effects on tendon stiffness adaptations and running economy.

## 1 Introduction

For endurance running or endurance events in general, it is classically accepted that the maximal oxygen uptake (VO_2_max) and the fractional utilization of the VO_2_max are key determinants of performance. A third major determinant of performance represents running economy (Jones 2016) that can be quantified as energy utilization at a given submaximal exercise intensity (Barnes and Kilding, 2015). According to Barnes and Kilding (2015) the factors affecting running economy are metabolic efficiency, cardiorespiratory efficiency, training, neuromuscular efficiency, as well as biomechanical efficiency. Moreover, compared to level running, uphill running causes more oxygen consumption (decrease in running economy), whilst downhill running less oxygen consumption (Lemire et al., 2021). Additionally, very comprehensive running such as an ultra-marathon can decrease the running economy in the course of the race (Scheer et al., 2018).

Concerning especially neuromuscular and biomechanical efficiency the largest proportion of energy consumption in running is based on the work done by muscles to lift and accelerate the body (Kram and Taylor, 1990). Additional work in running is performed by elastic connective tissue (e.g., tendons) using the stored energy with negligible metabolic cost. Besides the release of elastic energy, the length change of the previously stretched tendon influences the shortening velocity of the muscle-tendon-unit (MTU) and hence the force-length-velocity potential of the muscles. Thereby, an uncoupling of the muscle belly shortening behavior compared to whole MTU shortening can occur [i.e., tendon gearing; (Wakeling et al., 2011)]. In running, this tendon gearing effect of a slower muscle belly shortening velocity relative to the MTU velocity can be observed during the stance phase for the soleus or the vastus lateralis muscle and hence, contributes to increased muscle-specific efficiency (Bohm et al., 2021).

A possibility to change the functionality of a muscle or tendon (e.g., by changing its stiffness) within a warm-up is stretching with its various techniques (Kay, Husbands-Beasley, & Blazevich, 2015; Konrad, Stafilidis, & Tilp, 2017). However, studies that have investigated the acute effects of stretching on running performance and/or economy showed conflicting reports. While some studies have reported that a single bout of stretching has a positive effect on running performance/economy (Godges et al., 1989; Faelli et al., 2021), others have reported no effect (Allison et al., 2008; Damasceno et al., 2014) or even adverse effects (Wilson et al., 2010; Lowery et al., 2014).

An explanation for the contradictory body of evidence might be the applied approach to stretch multiple muscles before a running event (i.e., quadriceps, hamstrings, triceps surae, adductors). On the one hand, there is evidence that a more compliant quadriceps tendon and aponeurosis (Arampatzis et al., 2006; Bohm, Mersmann, Santuz, Schroll, et al., 2021) and also vastus lateralis muscle (Miyamoto et al., 2019) are associated with a better running economy in endurance athletes. Such a decrease in muscle and tendon stiffness could be induced by a single bout of stretching of the quadriceps (Konrad, Seiberl, Tilp, Holzer, & Paternoster, 2022). Accordingly, a single quadriceps stretching intervention should have a positive effect on running economy. On the other hand, a stiffer Achilles tendon (Gleim, Stachenfeld, & Nicholas, 1990; Arampatzis et al., 2006; Hunter et al., 2011; Bohm, Mersmann, Santuz, Schroll, et al., 2021) and a stiffer triceps surae muscle (Dumke et al., 2010) as well as increased (nonpathological) hamstrings tightness (Gleim et al., 1990; Jones, 2002; Trehearn & Buresh, 2009) are also associated with better running economy. Consequently, a single bout of stretching of these muscle groups (i.e., posterior chain), immediately before a running event, can make the MTU more compliant (Kay et al., 2015; Konrad et al., 2017), and will likely have a detrimental effect on running economy. Hence, it was speculated that undifferentiated and holistic pre-exercise stretching might simultaneously lead to both positive (especially in quadriceps) and negative (especially in hamstrings and triceps surae) effects on running economy, which would thus be counterbalanced and, hence, lead to unclear results (Allison et al., 2008; Konrad, Močnik, Nakamura, Sudi, & Tilp, 2021).

Moreover, to date, the vast majority of the studies on this topic used static stretching exercises that induce acute changes in muscle, but not in tendon stiffness (Kay et al., 2015; Konrad et al., 2017) although this has the potential to increase running economy. Alternatively, a single bout of proprioceptive neuromuscular facilitation (PNF) stretching with a moderate duration (i.e., 4 × 15 s) can reduce tendon stiffness (Kay, Husbands-Beasley, & Blazevich, 2015; Konrad, Stafilidis, & Tilp, 2017). Additionally, it is known that a single bout of stretching for more than 1 min decreases force production (Kay and Blazevich, 2012; Behm et al., 2016), and would therefore likely impair running economy as well. However, if sport-specific post-stretching dynamic activities (PSA) are included in the warm-up routine, a possible performance drop can be avoided according to most (Samson et al., 2012; Behm et al., 2016; Reiner et al., 2021) but not all studies (Konrad et al., 2022).

By assuming that an acute bout of PNF stretching including PSA can decrease the muscle stiffness (Reiner et al., 2021; Konrad et al., 2022) as well as tendon stiffness (Konrad et al., 2022) running patterns such as ground contact time, stride lengths, or stride frequency might be altered in runners resulting in acute changes in metabolic costs (i.e., running economy) (Mooses et al., 2021).

Therefore, the purpose of this study was to investigate if a single bout of moderate duration (4 × 15 s) PNF stretching of either the quadriceps or triceps surae muscles followed by PSA has an impact on running economy. In addition, spatio-temporal parameters (i.e., ground contact time, stride length, stride frequency), which might explain changes in running economy, will be determined. Based on the literature (Arampatzis et al., 2006; Konrad et al., 2022), we hypothesized that quadriceps stretching followed by PSA will lead to an improved running economy, whilst triceps surae stretching followed by PSA will result in a negative or no effect on running economy compared to using PSA solely without stretching.

## 2 Methods

### 2.1 Experimental design

**FIGURE 1:**
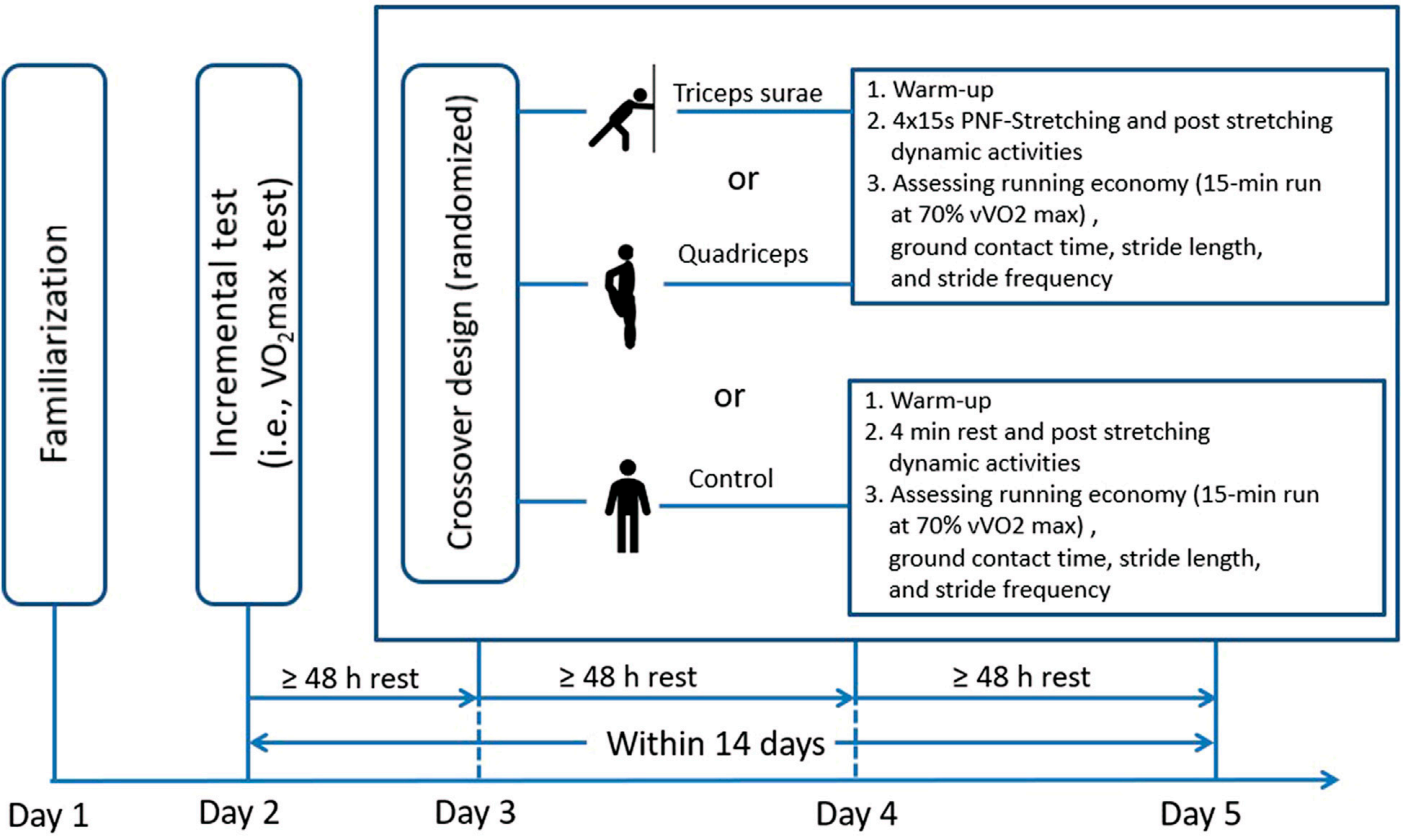
Schematic schedule of the study.

On the first day in the laboratory, participants were familiarized with the laboratory equipment (treadmill) and the test procedure (i.e., stretching exercises, PSA, incremental tests). Following the familiarization session, participants visited the laboratory another four times within a 14-day period with at least a 48-h rest between the test sessions. Participants were asked to be in a rested state (no hard workout 36 h before a measurement), to be hydrated, to have their last meal at least 3 h before the test (Hayes and Walker, 2007; Allison et al., 2008), to keep their nutrition constant throughout the 14 days, and to wear the same shoes throughout the tests (Allison et al., 2008). The measurements were undertaken at the same time of day (± 1 h), and the temperature and humidity in the laboratory were kept constant (21°C, 40% humidity) (Allison et al., 2008). On the second day, an incremental test was done to estimate the maximal oxygen consumption (VO_2_max) of the participants. On the third, fourth, and fifth day, participants were randomly assigned to either PNF stretching the triceps surae + PSA, PNF stretching the quadriceps + PSA, or no stretching + PSA (control condition) (see Figure 1). On testing days three to five, participants performed a standardized warm-up with 10 min treadmill running at 8 km/h (Damasceno et al., 2014). Subsequently, after the stretching interventions or control intervention the running economy test (15-min run on the treadmill at a velocity of 70% VO_2_max), including relevant biomechanical variables (ground contact time, stride length, stride frequency) was determined.

### 2.2 Participants

The primary outcome measure for the project was running economy. To the best of our knowledge, no study to date has analyzed the effect of PNF stretching (with a moderate duration) of a single MTU (triceps surae or quadriceps), followed by PSA, on running economy. Therefore, we used an explorative approach for the sample size calculation. Since we want to have the power to detect a medium to large effect, we estimated a required sample size of 18 participants for our study (repeated measures (within factors) ANOVA (three groups x three measures), partial ɳ^2^ = 0.1, α = 0.05, 1−β = 0.9, correlation among repeated measures = 0.5) using G*Power. Since male and female runners respond differently to a single bout of stretching before running (Mojock et al., 2011), we only included male participants in this study.

In accordance with a previous study (Damasceno et al., 2014) the inclusion criteria were recreational runners or triathletes, participating in endurance competitions, weekly running volume of more than 30 km, and training for at least 2 years without any interruptions. The exclusion criteria were pharmacological treatment, any type of neuromuscular disorder, dysfunction in the cardiovascular, respiratory, or circulatory system, and elite runner.

Consequently, we recruited 18 male trained runners/triathletes (age: 30.0 ± 6.1 years; weight: 75.4 ± 7.7 kg, height: 182.5 ± 4.6 cm). The average VO_2_max was 55.6 ± 6.7 ml kg^−1^ min^−1^ and the participants reported an average running mileage of 43.5 ± 12.6 km per week. The participants signed a written informed consent form, and ethical approval was obtained by the local ethical committee of the Technical University of Munich (762/20 S-KH) in accordance with the Declaration of Helsinki.

### 2.3 Procedures

#### 2.3.1 Incremental testing

To determine VO_2_max, an incremental test similar to a previous study (Damasceno et al., 2014) was performed on a motorized treadmill (Saturn 300/125, h/p/cosmos, Germany). The test started with a warm-up for 5 min running at 8 km/h, followed by an increase of 0.5 km/h every minute until full exhaustion. The stop criterion was when the participant was not able to maintain the velocity of the treadmill. Post-hoc, two out of the following three criteria were taken to confirm exhaustion and to determine VO_2_max: 1) an increase in VO_2_ between the consecutive stages of less than 2.1 ml/kg *min; 2) respiratory quotient exceeding 1.1; 3) exceeding the age-predicted (220 bpm—age) maximum heart rate (± 10 bpm) (Howely et al., 1995; Damasceno et al., 2014). A Cortex MetaLyzer 3B (CORTEX Biophysik, Germany) was used to measure gas exchange and flow volume, and hence to determine VO_2_ and VCO_2_ (carbon dioxide output). VO_2_ and VCO_2_ were averaged at 30 s intervals throughout the tests. Before all running tests (also running economy tests), the automated gas analysis system was calibrated using both ambient air and calibration gas (5% for CO_2_ and 15% for O_2_). A 3-L syringe was used to calibrate the volume sensor. A heart rate transmitter and heart rate monitor (Polar H10, Polar Electro, Kempele, Finland) were used to monitor heart rate.

#### 2.3.2 Running economy

To test running economy, subjects performed a 15-min run on the treadmill, reflecting a velocity of 70% VO_2_max determined during the second test day. A time of 15-min was taken since this is considered an appropriate duration to achieve a physiological steady-state (Barnes and Kilding, 2015). A running velocity of 70% VO_2_max is related to moderate intensity, below the respiratory compensation threshold (Esteve-lanao et al., 2005). The individual velocity of every subject was calculated from the relationship between the VO_2_ and the running velocities assessed during the incremental test (Yamaguchi et al., 2015). To calculate the running economy at this given speed, the VO_2_ was considered as an average value from 5 min of running at the steady-state in the last phase of the 15-min run.

#### 2.3.3 Ground contact time, stride length, and stride frequency

Ground contact time, stride length, and stride frequency were measured using an optical detection system (OptoGait, Microgate Corporation, Bolzano, Italy). Ground contact time is defined as the time span from the first contact of the foot until the take-off of the foot. Stride length is defined as the distance between the heel of two subsequent footprints of the same foot. Stride frequency can be determined from stride length and the constant velocity of the treadmill during the running economy trials. Ground contact time, stride length, and stride frequency were averaged throughout the same time window of the running economy measurements.

### 2.4 Stretching intervention and post-stretching dynamic activities

On test days 3–5, subjects were randomly assigned to either a single 4 × 15 s PNF stretching exercise of the triceps surae + PSA, quadriceps + PSA, or no stretching (4 min rest) + PSA (control condition). When the participants stretched their triceps surae, they were asked to perform this in a standing wall push position (Konrad and Tilp, 2014). For the quadriceps stretch, the participants were asked to stand upright on one leg and pull the ankle of the contralateral leg up to the maximum knee flexion (Stafilidis and Tilp, 2015). With both the quadriceps and the triceps surae stretch, the contract-relax PNF stretching technique similar to the stretching protocol of previous studies (Kay et al., 2015; Konrad et al., 2022) was applied. Participants were asked to stretch the target muscle (triceps surae or quadriceps) for 10 s, followed by a 5-s maximal contraction of the target muscle in the stretching position. This was done 4 times consecutively and resulted in an overall stretching/contraction duration of 60 s for each stretch. All stretches were performed on both legs and with a stretching intensity until the point of discomfort. During the control condition (no stretch) the participants were asked to rest for 4 min in a standing position.

Following the two stretching interventions and the control intervention, PSA according to the protocol of a previous study was performed (Samson et al., 2012; Konrad et al., 2022). Three different running-specific tasks were performed in a fixed order, immediately after the stretching exercises (i.e., triceps surae or quadriceps) or the 4 min break (i.e., control condition). The first task was a high knee run with a hip flexion of ∼90°. The second task was skipping. The third task was a “butt kick run”, where the heels should touch the bottom. All these tasks were performed twice over a 20-m distance (Samson et al., 2012). All the tasks were performed at a high speed (i.e., 7/10 on the visual analogue scale), and a break between the tasks of 30 s was scheduled. All interventions were supervised by the investigators.

### 2.5 Statistical analyses

SPSS (version 27.0, SPSS Inc., Chicago, Illinois) was used for all the statistical analyses. The Shapiro-Wilk test was used to test for the normal distribution of the residuals. If normally distributed, a one-way repeated measures ANOVA [three conditions = (triceps surae + PSA, quadriceps + PSA, control)] was used to test the effect of the stretching exercises + PSA on running economy and the related biomechanical parameters. If ANOVA was not applicable, we used the Friedman test. If the ANOVA or Friedman tests were significant, post-hoc paired t-tests or Wilcoxon signed-rank tests were performed, respectively. Cohen’s d was calculated following the suggestions of Cohen (1988). Thus, the effect size d was defined as 0.2, 0.5, and 0.8 for a small, medium, and large effect, respectively. The global level of significance was 5% for all tests.

## 3 Results

**FIGURE 2:**
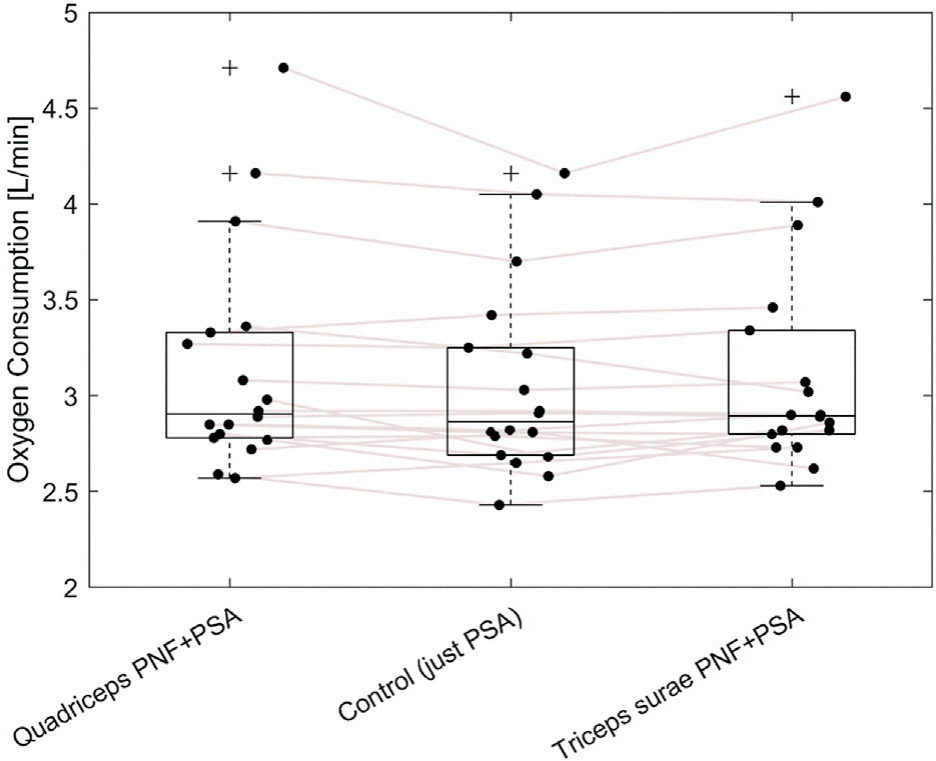
Boxplot diagram and individual results (black dots) of oxygen consumption of the three test conditions. PNF, Proprioceptive neuromuscular facilitation; PSA, Post-stretching dynamic activities. Black cross: Outliers, defined as more than 1.5 times the interquartile range away from the top of the box.

The average speed during the 15-min running economy runs was 11.3 ± 1.2 km/h, corresponding to individual running speeds at 70% VO_2_max. Mean values of all the tested parameters for the three conditions are shown in Table 1. Individual values are presented in Figure 2.

**TABLE 1:** Mean ± SD. Results for the parameters oxygen consumption, ground contact time, stride length, and stride frequency for the three crossover-design conditions. The two intervention conditions were quadriceps PNF stretching + post-stretching activation (PSA) and triceps surae stretching + PSA. Control condition was no stretching (4 min rest) + PSA (control).

	Quadriceps PNF stretching + PSA			Triceps surae PNF stretching + PSA			Control (4 min rest + PSA)		
Oxygen consumption (L/min)	3.14	±	0.58	3.11	±	0.54	3.05	±	0.49
Ground contact time (s)	0.286	±	0.031	0.287	±	0.034	0.290	±	0.033
Stride length (cm)	233.7	±	26.3	232.7	±	24.8	232.6	±	25.0
Stride frequency (stride/s)	1.34	±	0.07	1.35	±	0.07	1.35	±	0.07

Friedman test revealed no significant effect on oxygen consumption during the last 5 minutes of the 15-min steady-state run (*p* = 0.15; χ2 = 3.8). Moreover, one-way repeated measures ANOVA revealed no change in ground contact time (*p* = 0.52; F_2,16_ = 0.672; r = 0.077), stride length (*p* = 0.33; F_2,16_ = 1.194; r = 0.13), or stride frequency (*p* = 0.44; F_2,16_ = 0.870; r = 0.098).

## 4 Discussion

The purpose of this study was to investigate if a single bout of moderate duration (4 × 15 s) PNF stretching of the quadriceps or triceps surae muscles followed by PSA, has an impact on running economy compared to a control group that performed no stretching exercise but PSA. We assumed that isolated quadriceps PNF stretching + PSA will lead to a positive effect on running economy, whilst triceps surae PNF stretching + PSA will cause a negative or no effect on running economy. However, according to our findings, there was no difference between the interventions and control condition, indicating that additional stretching + PSA of these two specific muscle groups (quadriceps or triceps surae) has neither a positive nor a negative effect on running economy and the related biomechanical variables (i.e., ground contact time, stride length, stride frequency).

A few studies have already investigated the effects of a single bout of stretching on running economy and performance (i.e., time trial). Some studies have reported that static stretching has a negative effect on endurance performance (Wilson et al., 2010; Lowery et al., 2014) and energy expenditure (Wilson et al., 2010; Zourdos et al., 2012) similar to results in cycling (Esposito et al., 2011). These negative effects might be associated with an increase in ground contact time (Lowery et al., 2014). However, most of the studies have reported no changes in running performance (Allison et al., 2008; Mojock et al., 2011; Zourdos et al., 2012; Damasceno et al., 2014) or running economy (Hayes and Walker, 2007; Allison et al., 2008; Mojock et al., 2011; Damasceno et al., 2014; Yamaguchi et al., 2015), independent of the two stretching techniques used (static, dynamic). Nevertheless, some studies reported positive effects on running economy after a single bout of static (Godges et al., 1989; Faelli et al., 2021), dynamic (Faelli et al., 2021), or PNF stretching (Godges et al., 1989). Additionally, another study showed better running performance following a dynamic stretching intervention (Yamaguchi et al., 2015).

Bringing all the results together, there are conflicting reports in the literature about the effects of acute stretching prior to a running event. Except for the study of Godges et al. (1989), all the aforementioned studies stretched several MTUs prior to running tests, although it is known that stiff MTUs and tendons of the triceps surae (Gleim et al., 1990; Arampatzis et al., 2006; Hunter et al., 2011) or hamstring muscles (Gleim et al., 1990; Jones, 2002; Trehearn & Buresh, 2009) are advantageous for running performance/economy. Therefore, we assumed that a stretch of these MTUs that decreases MTU stiffness (Behm et al., 2016; Konrad and Tilp, 2020) will have a detrimental effect on running performance/economy, likely based on changes in the stretch-shortening cycle (i.e., longer ground contact time (Lowery et al., 2014)). However, the proposed advantage of a stiff Achilles tendon for running performance/economy would be impacted by running speed, where slower running velocities such as with slow, recreational jogging with their longer contact periods might actually benefit from more compliant tendons (Godges et al., 1989; Hayes and Walker 2007). The present results based on the running speeds of recreational runners and triathletes corresponding to 70% VO_2_max could not confirm the assumption of decreased MTU stiffness impairing running performance/economy.

Concerning the PNF stretching of the quadriceps muscles + PSA before running, we hypothesized that this will be advantageous since more compliant tendons were associated with a better running economy (Arampatzis et al., 2006; Bohm et al., 2021). The quadriceps muscle is especially active in the early stance phase, decelerating and supporting body mass and hence plays an important role during running. A decrease in quadriceps tendon stiffness achieved near significance (*p* = 0.06) in a previous study (Konrad et al., 2022) following the same PNF quadriceps stretching + PSA regimen used in the current study. Consequently, it was assumed that an increased tendon gearing (increase in ratio MTU vs.—muscle belly velocity) decreases muscle fascicle velocity resulting in an improved running economy. Just recently a further study (Bohm et al., 2018) showed that during the stance phase of running, the muscle fascicles of the vastus lateralis work almost isometrically and close to their plateau region of the force-length-relationship. The impact of an acute bout of moderate duration PNF stretching + PSA might be therefore insufficient to neither modify tendon gearing nor bring the fascicles further to the plateau region of the force-length relationship.

Some studies speculated that a possible negative effect of stretching the hamstrings and triceps surae cancelled out a positive effect of the stretching of the quadriceps muscles if performed in a combined stretching routine before a running event. As a result, no overall effect of stretching on the running economy could be observed (Allison et al., 2008; Konrad et al., 2021). However, our data showed that running economy was not different from the control condition (no stretching + PSA) neither following the quadriceps PNF stretching + PSA nor following the triceps surae PNF stretching + PSA, and hence, we cannot support the hypothesis of a counterbalancing effect. As a consequence of no significant changes in running economy, our results showed that the interventions did neither alter preferred stride frequency, stride length, nor ground contact time compared to the control condition. From a practical point of view, if stretching is part of an athlete’s warm-up routine, we recommend to use the PNF method with PSA. At least for submaximal endurance running in male recreational athletes no negative effects on running economy were found in our study.

One limitation of this study was the absence of an intervention condition in which only PNF stretching protocol of either the quadriceps or the triceps surae muscles is performed. Together with a further control condition (i.e., just rest without PSA) it would have been possible to get a clearer picture of muscle specific PNF stretching with and without PSA and its effect on RE. Future studies should take this into consideration.

## 5 Conclusion

To conclude, a single PNF stretching of the triceps surae or the quadriceps including PSA did not have a significant effect on running economy compared to PSA alone. The findings of the current study did not reflect our hypothesis that a single bout of PNF stretching of the triceps surae + PSA will result in a decrease in running economy since stiff triceps MTU (especially the tendon) was reported to be advantageous (Gleim et al., 1990; Arampatzis et al., 2006; Hunter et al., 2011). Additionally, we assumed a better running economy in the quadriceps condition, since a more compliant quadriceps MTU (especially the tendon) has been reported to be advantageous for running economy (Arampatzis et al., 2006; Bohm et al., 2021). Although a single PNF stretching exercise can decrease the overall MTU stiffness (Konrad et al., 2017) but also the tendon stiffness (Kay et al., 2015), this acute change did neither lead to beneficial (i.e., quadriceps) nor adverse effects (i.e., triceps surae) on running economy. Hence, future warm-up studies should investigate if other stretching techniques, which have the potential to change MTU stiffness [i.e., static stretching; (Konrad et al., 2017)] including PSA might lead to significant changes in running economy. Future training studies with the potential to chronically increase tendon stiffness [i.e., with isometric strength training (Albracht & Arampatzis, 2013)] or decrease tendon stiffness [i.e., with stretching training; (Konrad, Gad, & Tilp, 2015)] should be applied to test if such changes can induce a beneficial effect in running economy.

## Data Availability

The original contributions presented in the study are included in the article/Supplementary Material, further inquiries can be directed to the corresponding author.
